# Laparoscopic resection of a leiomyoma of the seminal vesicle

**DOI:** 10.4103/0970-1591.30272

**Published:** 2007

**Authors:** Bernard Lallemand, Philippe Busard, Frederic Leduc, Roland Vaesen

**Affiliations:** Department of Urology, Centre Hospitalier De L'ardenne, Libramont, Belgium

**Keywords:** Laparoscopic, leiomyoma, seminal vesicle

## Abstract

We present a case of a leiomyoma of the seminal vesicle that occurred in a 52-year-old man who presented with symptoms of bladder outlet obstruction. Prostate-specific antigen was within normal limit. Computed tomography scan and magnetic resonance imaging revealed a mass in the patient's right seminal vesicle. Laparoscopic excision of the seminal vesicle tumor was performed successfully. The patient was discharged from the hospital on the fourth postoperative day.

## INTRODUCTION

Primary mesenchymental tumors of the seminal vesicle are exceedingly rare lesions, with a few cases reported.[1] To our knowledge we present the first case of leiomyoma of the seminal vesicle resected by laparoscopy.

## CASE REPORT

**Figure 1:**
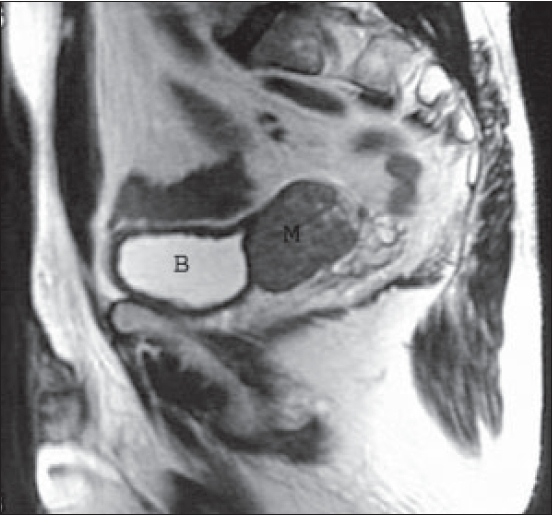
T2 weighted (TR/TE 4830/85) MR sagittal image reveals low signal intensity mass (M) arising from seminal vesicles. B, Bladder

**Figure 2:**
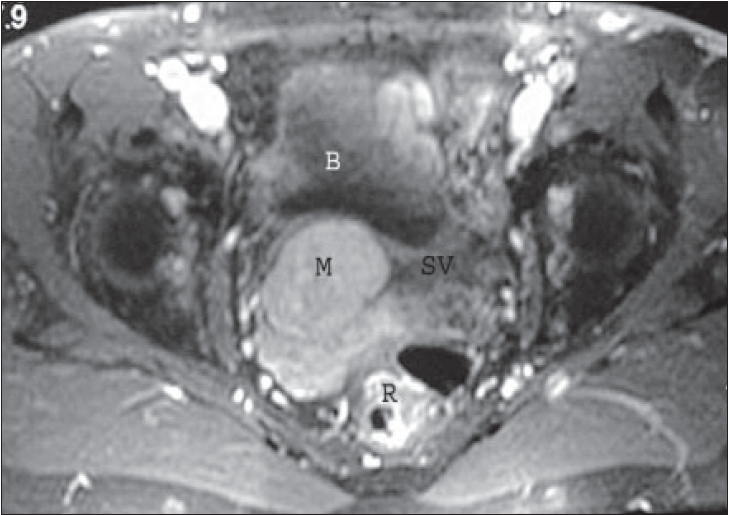
Postcontrast T1 weighted (TR/TE 430/10) MR axial image shows an homogenous enhancement of the mass (M). B, Bladder. SV, left seminal vesicle. R, rectum

A 52-year-old man presented with symptoms of bladder outlet obstruction for one month. A large prerectal soft mass was palpated on digital examination in the region of the prostate. Serum prostate-specific antigen (PSA) was within normal limit. Pelvic computed tomography scan showed a mass lesion in the right retrovesical region. The MRI revealed low signal intensity, well-marginated, ovoid mass 4×3cm in the right retrovesical located in the right seminal vesicle [Figure 1]. The left seminal vesicle was normal [Figure 2]. The MRI diagnosis was seminal vesicle fibroma.

The patient underwent video laparoscopic excision of the right seminal vesicle. The patient was operated under general anesthesia. The peritoneal cavity was filled with carbon dioxide through a Veress needle introduced transumbillicaly. The first 10-mm trocar was blindly introduced below the umbilic and the table was placed in the trendenlenburg position. Two 5-mm trocars were placed, under visual control, in the right iliac fossa and the left iliac fossa. A 5-mm trocar was inserted at the left paramedian level. The laparoscopic procedure was performed at an insufflation pressure of 12 mmHg. The retrovesical peritoneum was opened transversally and the right seminal vesicle was dissected to the vas deferens. The seminal vesicle artery was clipped and sectioned. The seminal vesicle was easily dissected from the posterior trigone of the bladder and the right ureter. The seminal vesicle was removed through the left paramedian trocar. The operative time was 70 min and blood loss was minimal.

The tumor was a well-circumscribed rubbery mass. Microscopically, the examination showed fascicles of spindle-shaped smooth muscle cells disposed in an interlacing pattern with abundant intervening hyalinized stroma. No cytologic atypia or mitotic figures were seen. Histopathological findings of the resected specimen were consistent with leiomyoma of the seminal vesicle.

The postoperative course was uneventful and the patient was discharged on the third postoperative day. RMN performed at one year follow-up revealed no recurrence of the tumor.

## DISCUSSION

Leiomyomas of the genitourinary tract are uncommon neoplasms that may arise from smooth muscles.[2] They have been described in kidneys, ureters, bladder, urethra, prostate, seminal vesicles, spermatic cord, testis, epididymis, penis and scrotum. The first case of leiomyoma of the seminal vesicle has been reported by Emmerich in 1910. The differential diagnosis of benign tumors of seminal vesicles includes cyst, fibradenoma, neuroma and angioendothelioma, mesenchyma and fibromuscular dysplasia. Malignant transformation (leiomyosarcoma) within a leiomyoma is extremely rare.

Seminal vesicle lesions are usually asymptomatic and can occasionally be an incidental finding on rectal examination. The most symptoms of a large symptomatic seminal vesicle mass are pelvic or perineal pain, dysuria, urinary frequency and bladder outlet or rectal obstruction.[3] The advent of modern imaging has vastly improved the detection of seminal vesicle lesions. Endorectal prostatic sonography has become routine and allows transrectal fine needle biopsy. A CT scan may delineate the general appearance of the structure of the pelvic cavity and raise the suspicion of seminal vesicular lesion. MRI, especially using endorectal coils, is superior to other modalities in determining the origin and the nature of retrovesical lesion.

Surgical excision of the seminal vesicular tumors is necessary; several open approaches have been tried. Recently, some authors reported video laparoscopy as an alternative approach to treat most seminal vesicle diseases.[4–5] We considered that laparoscopic management is a less invasive treatment, with minimal postoperative complication and shorter postoperative hospitalization.
